# The Role of Interleukin-8 (IL-8) in Treatment-Resistant Depression: A Review of Mechanisms, Biomarker Potential, and Therapeutic Implications

**DOI:** 10.3390/ijms262010092

**Published:** 2025-10-16

**Authors:** Katarzyna Aleksandra Lisowska

**Affiliations:** Department of Rheumatology, Clinical Immunology, Geriatrics and Internal Medicine, Faculty of Medicine, Medical University of Gdańsk, 80-211 Gdańsk, Poland; katarzyna.lisowska@gumed.edu.pl

**Keywords:** IL-8, inflammation, major depressive disorder, treatment-resistant depression, biomarker, ketamine

## Abstract

Treatment-resistant depression (TRD) remains a major clinical challenge, with a substantial proportion of patients with major depressive disorder (MDD) failing to respond to conventional antidepressant therapies. Increasing evidence suggests that dysregulation of immune signaling contributes to the pathophysiology of TRD. While proinflammatory cytokines such as IL-6 and TNF-α have been extensively studied, less is known about the role of chemokines such as interleukin-8 (IL-8). This review aims to synthesize current knowledge on the biological functions of IL-8, its involvement in neuroimmune mechanisms, and its potential as a biomarker and therapeutic target in treatment-resistant depression. Clinical and preclinical studies evaluating IL-8 levels in MDD and TRD patients were discussed with a focus on treatment response, neuroinflammatory pathways, and predictive modeling. Methodological factors affecting IL-8 measurement and interpretation were critically assessed. Even though clinical studies indicate that IL-8 levels may predict treatment response to antidepressants, including ketamine, findings are inconsistent, partly due to methodological variability, small sample sizes, and confounding factors such as comorbid medical conditions. Therefore, future longitudinal and multimodal studies are warranted to validate its utility in psychiatric precision medicine.

## 1. Introduction

Major depressive disorder (MDD) is a highly prevalent and disabling psychiatric condition, affecting more than 300 million people globally [1]. Despite the availability of multiple classes of antidepressants, approximately one-third of patients do not achieve adequate symptom remission, even after multiple treatment attempts [2]. This subset of patients is often classified as having treatment-resistant depression (TRD), a form of depression associated with higher morbidity, increased healthcare costs, and a greater risk of suicide.

The pathophysiology of TRD remains incompletely understood. Traditional models of depression have emphasized monoaminergic dysfunction—particularly involving serotonin, norepinephrine, and dopamine—but this framework does not sufficiently explain the clinical heterogeneity and treatment failures observed in TRD. Increasingly, evidence points to neuroinflammatory and immunological processes as critical components in the pathogenesis of depression, particularly its treatment-resistant forms [3].

Among the various immune-related factors implicated in MDD, cytokines—small proteins secreted by immune and non-immune cells—have emerged as key mediators of the brain–immune system crosstalk. Elevated levels of proinflammatory cytokines, such as interleukin-6 (IL-6), tumor necrosis factor-alpha (TNF-α), and C-reactive protein (CRP), have been observed in subsets of patients with depression and have been associated with poor treatment outcomes [4,5,6]. However, less attention has been paid to chemokines, a subset of cytokines that mediate the migration and activation of leukocytes.

TRD remains a major clinical challenge, affecting up to one-third of MDD patients and leading to considerable individual suffering, disability, and healthcare costs. Despite its prevalence, there are currently no reliable biomarkers that can predict treatment response or guide personalized therapeutic strategies. Growing evidence supports the involvement of immune dysregulation and chronic low-grade inflammation in the pathophysiology of TRD. Among pro-inflammatory cytokines, interleukin-8 (IL-8) is of particular interest due to its dual role as a mediator of immune activation and a regulator of neuroplasticity. Interleukin-8 (IL-8), also known as CXCL8, is a proinflammatory chemokine traditionally recognized for its role in recruiting neutrophils to sites of infection, which was identified in 1987–1988 [7,8,9,10]. Recent research suggests that IL-8 may play a more nuanced role in the central nervous system (CNS), influencing processes such as microglial activation, neurovascular function, synaptic plasticity, and response to antidepressant treatment. Preliminary findings indicate that IL-8 may serve as both a biomarker of treatment response and a potential therapeutic target in TRD [11,12,13]. Investigating the role of IL-8 in TRD is therefore of high importance, as it may provide insights into the biological underpinnings of poor treatment response and open avenues for novel, personalized, and potentially immunomodulatory treatment strategies.

This review aims to synthesize current knowledge on the biological functions of IL-8, its involvement in neuroinflammatory pathways, and its relevance to the pathophysiology and treatment outcomes of TRD. I highlight both preclinical and clinical evidence linking IL-8 to depression, evaluate its potential utility as a biomarker, and explore emerging therapeutic strategies that target IL-8-mediated mechanisms. This article summarizes publications from the last few years that have attempted to correlate IL-8 levels with TRD progression and treatment response. It also explains why studies from different centers yield varying results. There are also proposed directions for further research.

## 2. Biology and Functions of IL-8

IL-8 is a cytokine that belongs to the CXC subfamily [14]. It was primarily identified as a chemokine with the ability to attract neutrophils to sites of inflammation and stimulate their activity [15]. Presently, it is recognized as a multifunctional cytokine with a broad role in the immunological and neurological systems.

The genomic structure of the CXCL8 gene, which encodes IL-8, was described in 1989 [16]. Studies later showed that IL-8 is expressed in monocytes [17], macrophages [18], mast cells [19], endothelial [17,20], and epithelial cells [17,21] in response to inflammatory stimuli such as TNF-α [22,23], IL-1β [19], and microbial products, for example, lipopolysaccharide (LPS) [24]. Its biological effects are mediated through binding to two G-protein-coupled receptors: CXCR1 (IL-8RA) and CXCR2 (IL-8RB) [25]. These receptors are expressed on a wide variety of immune cells, including neutrophils, monocytes, and lymphocytes [26].

As mentioned above, IL-8 is a key chemoattractant and activator of neutrophils, playing an essential role in acute inflammation [15,27]. Its expression in different cells is initiated by pro-inflammatory cytokines TNF-α and IL-1β [19,22,23,28,29]. Neutrophils stimulated with IL-8 undergo chemotaxis [30], respiratory burst [31], and degranulation—mechanisms essential in host defense but also potentially damaging when dysregulated [32]. IL-8 also induces neutrophil extracellular trap (NET) formation in response to tissue damage [33], during systemic inflammatory response syndrome (SIRS) [34] or infection [35].

Beyond neutrophils, IL-8 modulates adhesion molecule expression, endothelial permeability, and angiogenesis [18,36]. According to some studies, IL-8 can also enhance endothelial cell survival by stimulation of anti-apoptotic gene expression (e.g., Bcl-2) [37]. IL-8 also contributes to tissue remodeling and maintenance of low-grade inflammatory states [38].

IL-8 expression is tightly regulated at the transcriptional and post-transcriptional level by nuclear factor-kappa B (NF-κB) [23,39,40], activator protein-1 (AP-1) [39,40], and hypoxia-inducible factors (HIFs) [41]. Importantly, IL-8 levels are also influenced by factors common in depression, such as stress [42], obesity [43], metabolic syndrome [44], and circadian disruption [45]. The increase in IL-8 in these conditions is most likely a consequence of the chronic inflammation that accompanies them, but its exact role is less well-known.

Early studies demonstrated that IL-8 can serve as a mediator of angiogenesis [18], a hallmark of chronic inflammation. Later, it was confirmed that IL-8, together with vascular endothelial growth factor (VEGF) and basic fibroblast growth factor (bFGF), is involved in the tubular morphogenesis in endothelial cells in response to stimulation with TNF-α [36]. In chronic pancreatic diseases, IL-8 was found to increase the expression of metalloproteinases (MMP), thus contributing to the invasiveness of human pancreatic cancer [46].

**Figure 1:**
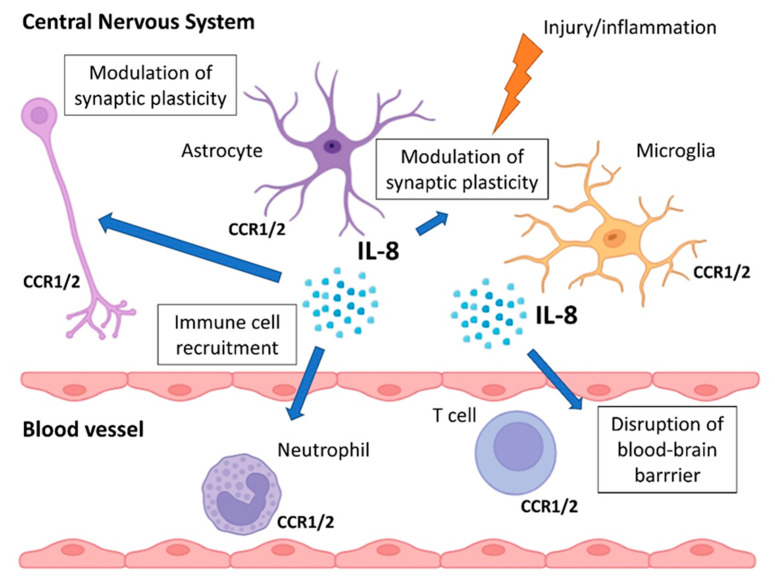
Role of IL-8 in CNS. Microglia, neurons, and astrocytes express IL-8 receptors (CCR1 and CCR2) and secrete IL-8 in response to injury or inflammation. IL-8 in the CNS recruits immune cells, disrupts the BBB, activates microglia, and modulates synaptic plasticity.

Although historically associated with peripheral inflammation, IL-8 and its receptors are also present in the CNS (Figure 1). Microglia [47] and astrocytes [48] can secrete IL-8 in response to injury [49] or inflammation [50,51]. Expression of IL-8 receptors, mainly CXCR2, has been demonstrated in neurons [52,53,54], astrocytes [55], and glial cells [56], suggesting potential autocrine or paracrine effects within the brain [48].

IL-8 is implicated in several neuroimmune processes, including recruitment of immune cells to the CNS, particularly under conditions of blood–brain barrier (BBB) disruption [57,58], activation of microglia, and modulation of synaptic plasticity [59], possibly via effects on glutamate signaling [60] and oxidative stress [61].

Emerging evidence points to IL-8 as a player in the pathophysiology of neuropsychiatric disorders, including major depression. Elevated peripheral IL-8 levels have been observed in subsets of patients with depression [11,13], although findings remain inconsistent. The precise mechanisms by which IL-8 contributes to affective symptoms are still under investigation but may include promotion of chronic low-grade inflammation [62,63], disruption of vascular integrity [64], or modulation of neuronal excitability [65].

## 3. IL-8 in Depression

Over the past two decades, accumulating evidence has implicated inflammation in the pathogenesis of major depressive disorder (MDD). Several meta-analyses have consistently demonstrated elevated levels of proinflammatory markers, including IL-6, TNF-α, and CRP, in subsets of patients with depression [4,5,6]. While these markers have been the primary focus of immuno-psychiatric research, interest in chemokines—particularly IL-8—has been steadily growing.

### 3.1. Evidence from Clinical Studies

Clinical studies examining IL-8 levels in patients with MDD have yielded mixed results (Table 1). Some cross-sectional investigations report elevated IL-8 concentrations in depressed individuals compared to healthy controls, suggesting the presence of low-grade systemic inflammation [11,66]. Others have observed lower or unchanged levels of IL-8, possibly reflecting heterogeneity in sample characteristics, depressive subtypes, comorbidities, or methodological differences such as sampling time and assay sensitivity [67,68].

Zhu et al. [68] demonstrated that serum IL-8 is lower in drug-free MDD patients compared to healthy people, and after treatment, it increases. Its Log10 levels were negatively correlated with the Hamilton Depression Rating Scale (HDRS) score in drug-free patients. Similar results were obtained by Mikova et al. [67] 20 years earlier. Meanwhile, Leighton et al. [66] and Dahl et al. [11] showed that MDD patients exhibit higher serum IL-8 than healthy controls. According to Islam et al. [69], IL-8 above 93.47 pg/mL was associated with the MDD state.

Although many authors report that high levels of IL-8 are characteristic of MDD, meta-analyses do not seem to confirm this. In 2010, Dowlati et al. [4] analyzed four different studies and proved that IL-8 was not consistently elevated in MDD; however, the authors saw increased levels in specific subgroups of patients. A couple of years later, Eyre et al. [70], who focused on chemokines in MDD, analyzed seven different studies and found no significant difference in IL-8 levels between MDD patients and healthy people. The authors also observed significant heterogeneity in included studies, which was associated with the mean age of the sample, gender, and assay used to analyze IL-8 levels.

### 3.2. Sex, Age, and Clinical Subtype Effects

Several studies suggest that sex and age may influence IL-8 expression in depression [69,71,72]. For instance, some research indicates that female patients with depression tend to exhibit higher IL-8 levels than males [69,71], potentially reflecting sex-specific immune responses or hormonal modulation of cytokine production [73]. However, higher IL-8 in women seems to be associated with a lower HDRS score [74]. It is important to remember that older age is also associated with increased baseline inflammation [75], which may confound the association between IL-8 and depressive symptoms.

### 3.3. Longitudinal and Interventional Studies

Longitudinal studies investigating the relationship between IL-8 and symptom trajectories remain limited. In some cohorts, baseline IL-8 levels have been found to predict subsequent severity [72,76] or persistence of depressive symptoms [76,77], while in others, IL-8 levels did not change significantly in response to standard antidepressant treatment [78] or even increased [79].

Interestingly, certain interventions—such as exercise, mindfulness-based therapies, and anti-inflammatory agents—have been associated with reductions in IL-8 levels, although these effects are modest and often nonspecific [80]. The heterogeneity of findings suggests that IL-8 may not be a universal marker of depression but may reflect a distinct inflammatory endophenotype present in a subset of patients. More recent findings suggest that IL-8 may play a modulatory rather than primary role in the inflammatory profile of depression, possibly interacting with other cytokines or being more relevant in specific clinical phenotypes (e.g., melancholic, atypical, or somatic depression) [81,82].

## 4. IL-8 in Treatment-Resistant Depression

Treatment-resistant depression (TRD), defined as the failure to respond to at least two adequate trials of antidepressant therapy, remains one of the most challenging forms of major depressive disorder. As evidence grows implicating neuroinflammation in TRD pathophysiology, increasing attention has turned to the potential role of inflammatory biomarkers in identifying treatment-resistant phenotypes and guiding personalized therapies. Interleukin-8 (IL-8), although less studied than IL-6 or TNF-α, has emerged as a potential indicator of immune dysregulation specifically linked to poor treatment outcomes.

### 4.1. Baseline IL-8 Levels and Treatment Response

Only a few studies have evaluated whether baseline levels of IL-8 are predictive of antidepressant treatment response. Syed et al. [83] were comparing IL-8 levels depending on the type of antidepressant treatment and response to therapy. Patients included in the study were receiving escitalopram or duloxetine. Some were undergoing cognitive behavior therapy (CBT). The authors did not observe changes in IL-8, although they noted that non-responders had higher levels of IL-1β and TNF. Meanwhile, Szałach et al. [13] not only demonstrated that serum IL-8 levels are higher in TRD patients than in healthy individuals but also showed that serum IL-8 levels above 19.55 pg/mL were associated with a 10.26-fold likelihood of developing TRD.

Kruse et al. [84] examined the influence of electroconvulsive therapy (ECT) on inflammation associated with depression, but they saw no difference in IL-8 level throughout the whole treatment. These findings challenge the simplistic notion that elevated inflammation is uniformly associated with worse outcomes, suggesting a more complex, possibly U-shaped or subtype-specific relationship between IL-8 and treatment efficacy.

### 4.2. IL-8 and Novel Antidepressant Interventions

Recent studies have explored the relationship between IL-8 and non-traditional antidepressants, particularly ketamine. Szałach et al. [12] recently demonstrated that serum IL-8 after ketamine infusion dropped in the first 4 to 24 h in TRD patients and remained at a lower concentration for the whole treatment. Kruse et al. examined the influence of ketamine treatment in TRD, and they demonstrated that increasing IL-8 was associated with decreasing HDRS score in women [74]. In men, the authors observed the opposite effect, which suggests that the role of IL-8 could be sex-dependent.

These findings suggest that IL-8 may not only serve as a predictor of treatment response but could also reflect early immunomodulatory effects of fast-acting antidepressants. The underlying mechanisms remain to be elucidated but may involve IL-8’s interaction with microglia, synaptic plasticity, and the glutamatergic system. Moreover, sex differences in IL-8 levels indicate connections with hormonal balance, which requires further research.

### 4.3. Confounding Factors and Methodological Issues

Despite promising findings, there is substantial heterogeneity across studies. Differences in sample size, depression severity, duration of illness, comorbidities (e.g., obesity, metabolic syndrome), and technical aspects (e.g., serum vs. plasma, ELISA vs. multiplex, timing of sampling) may contribute to these inconsistencies in IL-8 data [4,70]. Moreover, the directionality of IL-8 changes (higher vs. lower levels in TRD) may be influenced by whether studies examine unmedicated patients, partial responders, or individuals with chronic, refractory depression.

## 5. Mechanisms Linking IL-8 to Treatment Resistance

**Figure 2:**
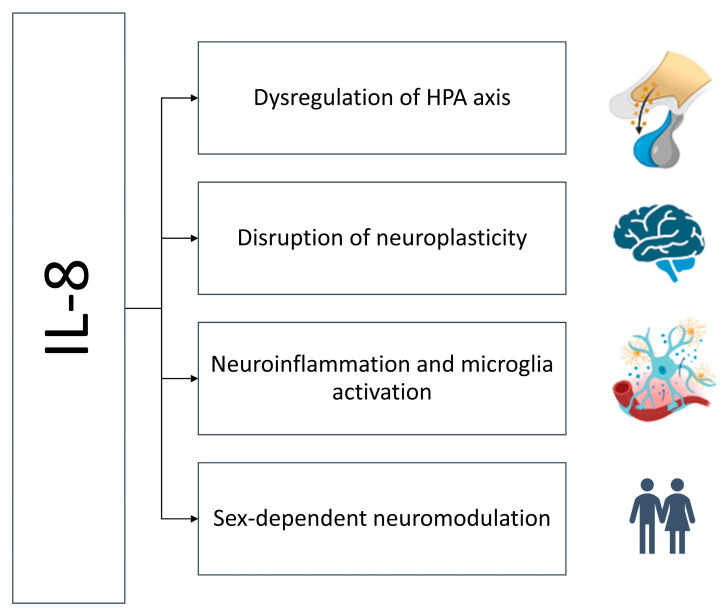
Mechanisms of IL-8-dependent treatment resistance. High levels of IL-8 activate the HPA axis, disrupt neurogenesis, activate microglia, and thus sustain neuroinflammation in a sex-dependent manner.

Although IL-8 has traditionally been viewed as a peripheral chemokine involved in neutrophil trafficking, emerging evidence suggests it may influence CNS function and contribute to the pathophysiology of TRD. Several interrelated mechanisms may explain how IL-8 could modulate antidepressant response (Figure 2).

### 5.1. Dysregulation of the Hypothalamic–Pituitary–Adrenal Axis

Inflammatory mediators such as IL-1, IL-6, or TNF interact with the hypothalamic–pituitary–adrenal (HPA) axis, leading to hyperactivation of cortisol signaling. In a state of health, acute stress induces the transmission of nerve signals from the cerebral cortex to the hypothalamus, where it stimulates the CRH secretion. CRH stimulates ACTH secretion by the pituitary gland, which results in the release of glucocorticoids (GCs) from the adrenal cortex. GCs act in an immunosuppressive manner on the immune cells, mainly by inhibiting the production of proinflammatory cytokines [85]. Sustained HPA activation is a hallmark of TRD and is associated with glucocorticoid resistance (GR) due to persistently high GC blood concentrations, resulting in reduced response of immune cells to GCs or a decrease in the threshold of hypothalamic sensitivity to pro-inflammatory cytokines secreted by these cells [3]. IL-8 may participate in this dysregulation, creating a feedback loop in which elevated IL-8 promotes HPA activity, and elevated cortisol further fuels immune dysfunction. There is not much information in the literature about the IL-8-HPA axis relationship. However, in 1992, Licinio and colleagues confirmed that the IL-8 gene is expressed in rat hypothalamus and hippocampus together with IL-1α, IL-1β, IL-1 receptors, and IL-1 receptor antagonist (IL-1RA) [86]. The presence of the expression of IL-8 and its receptors in the brain suggests that a neurological autocrine loop exists that could be activated in response to chronic inflammation [48,57,58]. Licinio et al. [86] suggested that the presence of IL-8 and its receptors in the hypothalamus and hippocampus could be relevant for the regulation of stress-related neuroendocrine function. Their suggestion was proven by the example of newborns; Yektaei-Karin et al. [87] demonstrated that stress associated with assisted delivery increased IL-8 and cortisol levels in umbilical arterial blood samples.

### 5.2. Neuroinflammation and Microglial Activation

The presence of pro-inflammatory factors in the cerebrospinal fluid (CSF) [88], the association of pro-inflammatory cytokine levels with depressive symptoms [89], and finally the reduction of depressive symptoms after the administration of anti-inflammatory drugs [90] are evidence that neuroinflammation plays a significant role in the pathogenesis of depression, especially TRD [91]. Microglia and astrocytes play an important role in the development of neuroinflammation.

The phenotype of microglia depends on their activation status [92]. Under chronic stress or in inflammatory states, microglia become activated by IL-1β, TNF-α, or LPS and secrete cytokines and chemokines, including IL-8 [47]. In turn, IL-8 can interact, for example, with cholinergic neurons in the brain and modulate their excitability by closing calcium channels [65]. Elevated IL-8 may also amplify neuroinflammation by recruiting peripheral immune cells across the BBB and stimulating them to produce more proinflammatory cytokines, thus contributing to sustained neuroimmune dysregulation [58]. Studies have also shown that IL-8 can modulate the activity of glial cells [93], enhance neuronal survival, and stimulate the proliferation of glial cells and astrocytes [94].

### 5.3. Disruption of Neurogenesis and Neuroplasticity

Neurogenesis, particularly in the hippocampus, is essential for effective antidepressant response [95]. A meta-analysis by Videbech and Ravnkilde [96] revealed that patients with MDD are characterized by decreased hippocampal volume. Currently, there is no direct evidence for the role of IL-8 in the process of neurogenesis. However, some authors suggest that it could inhibit neurogenesis by several mechanisms, including increasing oxidative stress, reducing the brain-derived neurotrophic factor (BDNF), and impairing neuronal differentiation [97].

BDNF plays a significant role in neuronal survival and differentiation during development and maintains high expression levels in different parts of the human adult brain, including the hippocampus [98]. Moreover, BDNF also exerts anti-inflammatory properties towards microglia; studies showed that it inhibits the production of IL-6 or TNF-α in response to LPS stimulation in primary glial cell cultures in mice [99]. The relationship between IL-8 and BDNF was noticed in newborns a couple of years ago, when it was demonstrated that newborn children diagnosed with neonatal encephalopathy have increased plasma levels of IL-8 and decreased BDNF levels, compared with healthy children [100]. Similar observations were also made in patients with schizophrenia [101] and bipolar disorder (BD) accompanied by alcohol use disorder (AUD) [102]. A meta-analysis by Cavaleri et al. [103] showed that lower peripheral and central BDNF levels characterize MDD patients as well. However, whether it is IL-8 that contributes to the reduction in BDNF is presently unclear.

Another factor influencing neuroplasticity is nerve growth factor (NGF), which is involved in modulating the cholinergic system, especially in the hippocampus [104]. Changes in NGF expression have already been associated with BD, AUD, schizophrenia, and depression [105]. However, in the case of depression, the data on NGF levels are inconclusive. Some authors demonstrated that patients with depression had lower NGF compared with healthy people [106], which increased during successful treatment [107,108], while others saw no significant difference or correlation with treatment [109]. Experimental studies show that IL-8 stimulates NGF production in astrocytes during brain injury in response to BBB disruption [49], which would suggest that it has a protective role.

Excessive production of reactive oxygen species (ROS) that increases apoptosis of nerve cells is another well-known mechanism disrupting neurogenesis [110,111]. In the brain, apart from neutrophils, other sources of ROS may be microglia [111] and astrocytes [112]. As mentioned earlier, so far, it has not been proven that IL-8 stimulates any of these cells to produce ROS. However, neutrophils activated by IL-8 release proinflammatory cytokines that could stimulate astrocytes and glial cells to produce cytokines [112,113] and ROS and thus contribute to inhibiting neurogenesis [113].

Even though some of these observations show that chronic elevations in IL-8 could create a microenvironment hostile to synaptic remodeling and emotional regulation, the contradictory nature of these reports, as well as the varying effects of antidepressant therapy on IL-8 levels, demonstrates that the functional significance of IL-8 in neurogenesis requires further investigation.

### 5.4. Sex-Specific Immunomodulation

Recent findings [72,74] suggest that the effects of IL-8 on treatment response may vary by sex. For example, in women with TRD, an increase in IL-8 following ketamine infusion was associated with clinical improvement, whereas no such association was observed in men. This points to hormone-mediated immunoregulation and the possibility of sex-specific IL-8 signaling pathways in depression. Immunity between females and males differs; female hormones like estrogens, progesterone, and prolactin are involved in the regulation of homeostasis between a range of immune cells [114]. Regarding cytokine/chemokine balance, the studies by Aulock et al. [115] showed that a higher concentration of several proinflammatory cytokines, including IL-8, characterizes blood from men. Several other authors saw that serum IL-8 is higher in women [116]. Moreover, it has also been observed that, especially in women, high levels of circulating IL-8 are positively correlated with a higher sensitivity to pressure pain [117]. Furthermore, high concentrations of IL-8 in the cerebrospinal fluid accompany fibromyalgia, a typically female disorder that is often accompanied by depression [118]. Another female disorder, endometriosis, is associated with high IL-8 tissue expression that also correlates with patient-reported pain level [119].

These observations show that when analyzing the level of cytokines or chemokines, the patient’s gender should be taken into account, and in the case of women, even the phase of the menstrual cycle should also be taken into account.

## 6. IL-8 as a Potential Biomarker in Psychiatry

The pursuit of reliable biomarkers in psychiatry has intensified over the past decade, driven by the need to improve diagnosis, stratify patients, and guide treatment decisions. Among immune markers under investigation, interleukin-8 (IL-8) has emerged as a candidate due to its links with neuroinflammatory processes, treatment response, and potential for individualized risk profiling, particularly in treatment-resistant depression (TRD).

### 6.1. Requirements for an Effective Biomarker

The ideal biomarker in psychiatry should meet several key criteria. First of all, biological plausibility, which means that it must reflect underlying pathophysiology relevant to the disorder [120]. The second important thing is reliability and reproducibility. Results must be consistent across settings, platforms, and populations. Thirdly, a biomarker should be characterized by sensitivity and specificity, which means that it should discriminate between disease states (e.g., responders vs. non-responders). It must inform diagnosis, prognosis, or treatment decisions [121]. Finally, it should be characterized by feasibility, which means a low cost, non-invasive sampling, and easy integration into clinical workflows. Interleukin-8 (IL-8), due to its role in immune signaling, BBB permeability, and neuronal function, meets several of these biological criteria. However, its utility in clinical psychiatry remains exploratory [122].

### 6.2. Strengths and Limitations of IL-8 as a Marker

IL-8 as a potential marker of depression would have many strengths. According to different authors, it is involved in depression pathophysiology; it plays a role in neuroinflammation, synaptic plasticity, and stress response. Also, several studies link IL-8 levels with antidepressant response, particularly to ketamine [12,74], which points to its associations with treatment outcomes. Additionally, recent findings suggest predictive value in women [72,74], offering a route to personalized interventions. And so, even though many of the reports discussed here show that IL-8 may play an important role in depression, especially treatment-resistant depression, IL-8 as a potential marker of TRD or treatment response to antidepressants is still in question due to several limitations related to its measurement. The most important limitation is the inconsistency of findings; some studies report elevated IL-8 in MDD and TRD, others find reduced or unchanged levels [123]. Also, IL-8 is characterized by low specificity as it is elevated in many somatic conditions (e.g., infections, cancer, obesity). Evaluating IL-8 concentrations in patients’ blood is also problematic. Due to the methodology used, there is no consensus on what concentration range is truly pathologically significant.

### 6.3. Methodological Issues

Biomarker research in psychiatry, including IL-8 studies, faces several methodological challenges. One of the most important is the sample type. IL-8 levels may differ significantly between serum and plasma due to platelet degranulation during clotting. Meanwhile, as shown in Table 1 and Table 2, different studies use different types of biological material, which may influence the IL-8 concentration. Another important issue is assay variability, which is also presented in both Tables. Results differed depending on whether ELISA, CBA, Luminex, or other multiplex platforms were used. The same set of cytokines/chemokines can be measured alone using ELISA or in combination with other proteins using multiplex technologies. They can be analyzed by spectrophotometry or flow cytometry. Platchek et al. [124] compared three of the most common proinflammatory cytokines: IL-1β, IL-6, and TNF, which are also associated with depression, in the same samples but using different technologies and demonstrated that they can differ in sensitivity, dynamic range, and robustness. Similar observations were made by Zhang et al. [125]. Their research shows that consistency in matrix selection is crucial if you want to demonstrate the potential of a cytokine or chemokine as a biomarker.

The time of day when the blood sample is collected is also essential, because IL-8 changes according to a circadian rhythm [40]. Of course, there are many other confounding variables, like medications (e.g., NSAIDs, corticosteroids, antipsychotics), lifestyle factors (smoking, alcohol, physical activity), comorbid conditions (metabolic syndrome, autoimmune disease), or hormonal influences (menstrual cycle, menopause), which can mask or mimic disease-specific IL-8 signals.

### 6.4. Potential for Predictive Modeling

The studies described in this article demonstrate significant limitations of current research on IL-8 in TRD, stemming from heterogeneity, small sample sizes, and lack of standardization. This underscores the need for interventional and longitudinal studies, as well as studies using multi-omics approaches and brain imaging [122]. Despite its limitations, IL-8 may gain diagnostic or prognostic value when used as part of multivariable models, for example, as part of the cytokine panels combining IL-8 with IL-6, CRP, TNF-α, and BDNF. It could also be used in treatment prediction algorithms, especially in stratifying patients for anti-inflammatory or glutamatergic interventions (e.g., ketamine).

Another option is to use machine learning (ML) models trained on clinical, biochemical, and neuroimaging data [126]. A remarkable advantage of ML in neuroimaging is the anatomical measurement and quantification of changes and disease patterns, which not only enables the rapid differentiation of acute from chronic conditions but also allows for tracking of imaging changes over time. The usefulness of neuroimaging is no longer limited to tumors, lesions, inflammation, and neurodegenerative diseases. A growing number of publications are emerging, demonstrating that various imaging techniques can also be useful in diagnosing and differentiating depression [127]. Also, a recent study by Vu et al. [128] demonstrated the use of ML to predict depression based only on socioeconomic, demographic, and health-related factors like physical activity, smoking, or drinking, which are self-reported, introducing potential inaccuracies, which suggests that adding metrics such as inflammatory markers, including IL-8, could significantly enhance ML models.

## 7. IL-8 as Therapeutic Target in Depression?

Although many clinical observations and studies in animal models indicate a significant role of IL-8 in maintaining the chronic inflammatory process in the CNS in patients with depression, we are still in the very early stages of understanding whether its contribution is significant enough to be used as a therapeutic target. Another significant problem is the variability of the obtained results; while some authors believe that high IL-8 levels are a marker of TRD, many others believe that higher IL-8 levels may be more beneficial, mainly due to their correlation with a good response to antidepressant treatment. Without strong evidence of the involvement of IL-8 in the development of depression, especially drug-resistant depression, the use of drugs modulating its amount or activity remains questionable. Therefore, before initiating studies that directly manipulate IL-8 levels, it is worthwhile to first examine non-pharmacological effects (training, nutrition) and targeted methods (e.g., antibodies) to see whether IL-8 modulation improves depressive symptoms. Also, it would be useful to initiate a personalized approach (precision psychiatry) that includes IL-8 as part of biomarker panels and predicts response.

## 8. Conclusions

**Figure 3:**
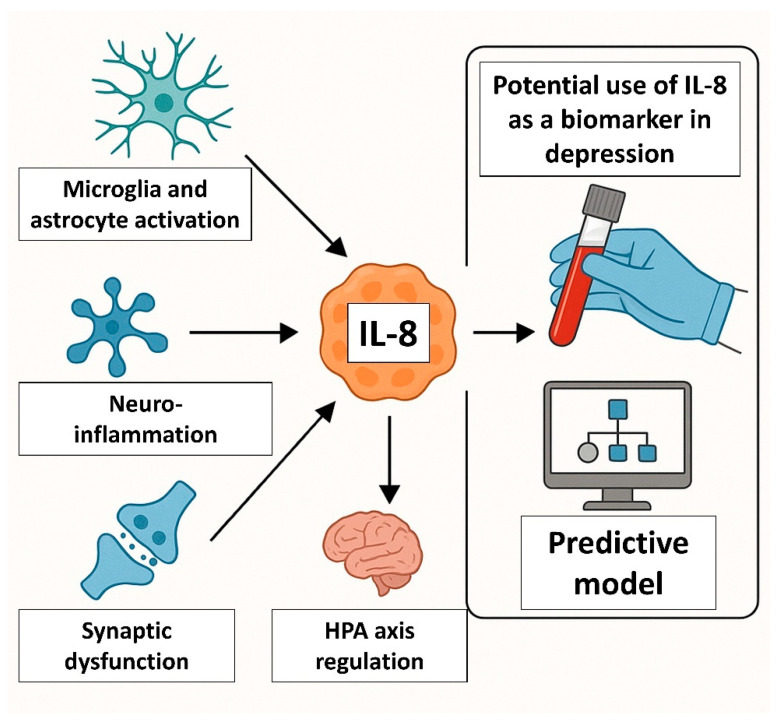
IL-8 as a potential biomarker of depression. IL-8 could serve as a screening tool to identify patients with immune-inflamed subtypes of depression and be a good predictor of response to antidepressants, particularly those with anti-inflammatory or glutamatergic mechanisms.

If validated in large-scale studies, IL-8 may serve several roles in psychiatric care (Figure 3). It could serve as a screening tool to identify patients with immune-inflamed subtypes of depression. IL-8 seems to be a good predictor of response to antidepressants, particularly those with anti-inflammatory or glutamatergic mechanisms. First studies also show its potential as a monitoring biomarker for early treatment effects (e.g., post-ketamine modulation). However, there are several obstacles to implementing IL-8 as a clinical biomarker, including pre-analytical variability (sample type), assay heterogeneity used to determine IL-8 level (ELISA vs. multiplex platforms yield different results), influence of comorbidities (obesity, infections, autoimmune disorders may affect IL-8 independently of depression), or even sex and age effects, which require stratified analyses.

To fully understand the role of IL-8 in treatment-resistant depression, future studies should begin by examining IL-8 levels in relation to different TRD phenotypes. Assessing IL-8 concentrations in serum or cerebrospinal fluid across various subtypes of TRD could reveal whether specific inflammatory profiles correlate with resistance to certain treatments, such as pharmacotherapy, electroconvulsive therapy, or novel biological interventions. This stratification could lead to more personalized and effective treatment approaches.

Additionally, there is a pressing need to investigate the molecular mechanisms by which IL-8 contributes to TRD. Preclinical studies—both in vitro and in vivo—could explore how IL-8 affects neuroinflammatory pathways, such as microglial activation, blood–brain barrier integrity, or the regulation of neurotrophic factors like BDNF. Understanding these mechanisms could clarify how IL-8 influences neuroplasticity and mood regulation, providing a biological basis for its potential role in depression pathogenesis.

Further research should also address how IL-8 interacts with other cytokines and inflammatory mediators. Exploring the synergistic or antagonistic effects of IL-8 in relation to pro-inflammatory factors such as TNF-α, IL-1β, and IL-6 could help define whether IL-8 acts as an upstream regulator, downstream effector, or part of a broader cytokine network. Such studies would provide a more integrated view of the neuroimmune dysregulation present in TRD.

Moreover, given the growing interest in anti-inflammatory strategies for depression, future work should explore therapeutic options targeting IL-8 or its signaling pathways. This could include the use of small-molecule inhibitors, monoclonal antibodies, or receptor antagonists targeting IL-8 receptors (CXCR1 and CXCR2). Evaluating the antidepressant efficacy of such agents in both clinical and preclinical models may reveal novel treatment avenues, particularly for patients unresponsive to conventional antidepressants.

Finally, longitudinal studies are needed to monitor changes in IL-8 levels over time in patients with TRD. Tracking IL-8 during various phases of illness and treatment could help determine whether its levels serve as a predictive marker of treatment response or relapse. Such studies could also provide insights into the temporal dynamics of neuroinflammation and inform the timing of targeted interventions.

A multi-dimensional research approach—encompassing phenotypic stratification, mechanistic studies, cytokine interaction mapping, targeted therapies, and longitudinal analysis—will be essential to clarify its role and therapeutic potential in TRD.

## Figures and Tables

**Table 1:** IL-8 levels in MDD.

Author (year)	Type of Research	Number of Participants	IL-8 Level	Material/Measuring Method
Islam et al. (2022) [69]	A case–control study	63 MDD/94 HC	IL-8 higher in MDD	Serum/ELISA
Zhu et al. (2022) [68]	Comparison of groups	87 MDD/101 HC	IL-8 lower in MDD patients	Serum/CBA
Leighton et al. (2018) [66]	Comparison of cytokine levels between groups	80 MDD/40 HC	IL-8 higher in MDD patients	Plasma/ELISA
Eyre et al. (2016) [70]	A meta-analysis	316 MDD/265 HC	No difference in IL-8	Serum or plasma/ELISA or multiplex
Dahl et al. (2014) [11]	Comparison of cytokine levels between groups	50 MDD/34 HC	IL-8 higher in MDD patients	Plasma/multiplex
Dowlati et al. (2010) [4]	A meta-analysis	205 MDD/177 HC from 4 studies	No difference in IL-8	Serum or plasma/ELISA or multiplex
Mikova et al. (2001) [67]	Comparison of cytokine levels between groups	47 MDD/20 HC	IL-8 lower in MDD patients	Serum/ELISA

CBA—Cytometric Bead Array, ELISA—Enzyme-Linked Immunosorbent Assay, HC—healthy control.

**Table 2:** Changes in IL-8 in TRD.

Author (Year)	Number of Participants	Type of Treatment	Relationship Between IL-8 Level and Response to Treatment	Material/Measuring Method
Szałach et al. (2025) [12]	18 TRD	Ketamine	Response to treatment associated with decreasing IL-8 levels	Serum/CBA
Kruse et al. (2021) [74]	46 TRD	Ketamine	Response to treatment associated with increasing IL-8 in women and decreasing IL-8 in men	Plasma/multiplex
Kruse et al. (2018) [84]	29 TRD	Electroconvulsive therapy	No changes in IL-8 during treatment	Plasma/multiplex
Syed et al. (2018) [83]	171 MDD	Escitalopram, duloxetine, CBT	No changes in IL-8 during treatment	Plasma/multiplex

CBT—Cognitive Behavior Therapy.

## Data Availability

No new data were created or analyzed in this study.

## Abbreviations

The following abbreviations are used in this manuscript:

CBA	Cytometric Bead Array
ELISA	Enzyme-Linked Immunosorbent Assay
HDRS	Hamilton Depression Rating Scale
HPA	Hypothalamic–Pituitary–Adrenal
IL	Interleukin
MDD	Major Depressive Disorder
TRD	Treatment-Resistant Depression
